# Recent Research Provides Significant New Information about Predisposing Factors, Diagnostic Practices, and Treatment of Carpal Tunnel Syndrome

**DOI:** 10.3390/jcm11185382

**Published:** 2022-09-14

**Authors:** Jorma Ryhänen

**Affiliations:** Department of Hand Surgery, Helsinki University Hospital, University of Helsinki, FI-00029 Helsinki, Finland; jorma.ryhanen@hus.fi

This current Special Issue of *JCM* will highlight some of the latest studies on carpal tunnel syndrome (CTS). This common upper extremity compression neuropathy can lead to a permanent lack of sensation in the median nerve area of the hand as well as thenar muscle atrophy. In addition to unpleasant symptoms, the disease can lead to incapacity for work, sick leave, and disability.

There are many aspects of CTS that are still unclear. Through active research, we generate a better overall picture of this disease’s etiology, risk factors, implications, and effective treatment options.

In the review of Zimmerman et al. [1], the pathophysiological aspects of CTS relating to diabetes are summarized. This comprehensive article combines current data and explains the increased risk of CTS in individuals with diabetes.

One can quickly think that only local wrist-level compression of the median nerve causes problems in CTS, but the picture is more comprehensive. When analyzing psychotropic medication in CTS patients in a large national register study, Dahlin et al. [2] found that surgically treated individuals with a nerve compression disorder have a higher risk of impaired psychological health.

Additionally, one study in this Special Issue states that patients with CTS have signs of changing central pain mechanisms. This might be associated more with psychological factors than central pain processing in people with focal nerve injuries [3].

Some anatomical risk indicators for CTS have been pointed out, such as body mass index, as found in the extensive birth cohort study by Lampainen et al. [4].

The diagnosis of CTS faces some challenges. Diagnosis is usually made from right anamnesis, clinical symptoms, and electromyography (EMG) findings. EMG studies are routinely used as a diagnostic tool of CTS, but these studies also contain sources of uncertainty. In the study of Sasaki et al. [5], a robust negative correlation between sensory nerve conduction velocity and distal motor latency was found, meaning that the severity classifications do not always correctly reveal the severity of CTS.

Ultrasound (US) has also been used as a diagnostic tool for CTS. In the study by Song et al. [6], most electrodiagnostic measurements revealed substantial correlations with roentgenographic and ultrasonography features. The electrodiagnostic severity was also correlated with imaging characteristics and wrist X-rays, and US may help diagnose CTS as a supplement to electrodiagnostic studies.

The article of Heiling et al. [7] concludes that diagnosis of CTS in diabetic patients should mainly be based upon specific anamnestic information and clinical findings. They point out that electrodiagnostic testing and nerve US results must be interpreted carefully, and other factors must also be considered.

In the article by Watanabe et al. [8], a specific hand drawing test was used to screen CTS patients, and was found to be effective for this purpose.

The differential diagnosis of CTS to more proximal median nerve problems is essential and should be carefully considered when deciding on the proper treatment. The review of Löppönen et al. [9] clarifies that proximal median nerve compressions (PMNC) should be seen as a continuum of mild to severe nerve lesions along a branching median nerve, thus producing variable symptoms. The diagnosis should be based on a more thorough understanding of anatomy and clinical examination. In PMNC, intervention should be planned according to each patient’s condition. To point out the complexity of causes and symptoms, PMNC should be named proximal median nerve syndrome.

How about the conservative treatment of CTS? In this Special Issue, Karjalainen et al. [10] review the present evidence of non-surgical treatments for CTS. These practices are diverse, and an unambiguously superior method is not clear. They found that many studies propose small short-term advantages of certain treatments, but evidence of their long-term benefits is weak. The article concluded that “research in this area should focus on establishing the value of each treatment instead of comparing various treatments with uncertain benefits”.

Surgery is reserved for those patients that do not attain a satisfactory symptom state by non-operative means, but what is the best method?

Segal et al.’s [11] review describes the current practice of carpal tunnel release (CTR) using the wide-awake, local-anesthesia, no-tourniquet (WALANT) technique. This is associated with substantial cost savings and a faster workflow. It can be safely implemented in an outpatient clinic with less use of resources. The authors conclude that WALANT surgery is able to achieve standard or better postoperative pain control and satisfaction for patients.

Another widely discussed technical issue is whether to treat CTR using endoscopic (ECTR) or open methods. Both surgeries are widely utilized, and there are no significant differences in the long-term postoperative results [12]. However, ECTR has certain advantages, such as less scarring and a shorter recovery period. This facilitates an earlier return to daily life activities. However, there are concerns about the potential for transient or permanent nerve injury. In the study of Yamamoto et al. [13], the annual open and endoscopic carpal tunnel release figures of Japan were documented, and their trends, gender differences, age distributions, and regional variations were analyzed. The results show that almost 40,000 CTRs were performed annually in Japan, and open CTR was implemented nearly four times more often than endoscopic CTR.

When the diagnosis and treatment of CTS are too late for the nerves to recover, or there is a surgical complication or failure of initial surgery, there might be a need for some late reconstructive surgeries. These should be performed by experienced hand surgeons. The approach and management of failed CTR are reviewed in the comprehensive article by Pripotnev et al. [14]. These patients can be categorized into persistent, recurrent, and new symptom groups. The operative treatment of revision cases included the proximal exploration of the median nerve, the re-release of the transverse retinaculum and scar, the evaluation of the nerve injury, the treatment of secondary sites of compression, and possible supplementary procedures. In addition, ulnar nerve release with neurolysis in the Guyon’s canal is recommended.

Researchers and clinicians from different fields sometimes look at CTS only from their narrow perspective of epidemiology, diagnostics, or surgical treatment. However, it would be useful to have a broader view of the matter. This Special Issue offers an interdisciplinary approach to the CTS problem from different perspectives. I hope the reader will take new inspiration from it for further research, aiming to achieve the best patient care.
